# Opioid Antagonists May Reverse Endogenous Opiate “Dependence” in the Treatment of Self-Injurious Behavior

**DOI:** 10.3390/ph4020366

**Published:** 2011-01-28

**Authors:** Curt A. Sandman, Aaron S. Kemp

**Affiliations:** Department of Psychiatry and Human Behavior, University of California, Irvine School of Medicine; 333 The City Blvd, Orange, CA 92868, USA; E-Mail: akemp@uci.edu

**Keywords:** opiates, endorphin, opiate blockers, POMC, naltrexone, self-injurious behavior, SIB

## Abstract

Self-injurious behavior (SIB) is a primary reason that individuals with neurodevelopmental disabilities (NDD) are either retained in restrictive environments or are administered psychotropic medication. There are no known causes and no universally accepted treatments for this complex behavior among individuals with NDD. There is developing evidence, however, that individuals exhibiting SIB have a disturbance of the opiate-mediated pain and pleasure system. One hypothesis is that SIB reflects insensitivity to pain and general sensory depression (hypoalgesia), perhaps related to chronic elevation of endogenous opiates. For instance, many self-injurious individuals do not exhibit the usual signs of pain after their “injurious” behavior. Moreover, for some individuals the addictive properties of elevated endogenous opiates (euphoria) may be responsible for maintaining their SIB. In this perspective, SIB may be viewed as an addiction because it supplies the “fix” for tolerant, down-regulated opiate receptors. Reports that levels of endogenous opiates at rest and after SIB episodes predict positive responses to opiate blockers (e.g., naltrexone) provide further support for opiate-mediated SIB and form the basis for a rational treatment strategy. Although the long term effects of opiate blockers on SIB are unknown, reduction in SIB following acute treatment provides support that a specific biological system may be dysregulated in a subgroup of patients. It is concluded that naltrexone produces a clinically significant reduction in the serious and life-threatening behavior of self injury for individuals who have not been responsive to any other type of treatment. Several suggestions and cautions are provided for regimens of naltrexone treatment of SIB.

## 1. Introduction

Despite considerable research effort, self-injurious behavior (SIB) continues to be a primary reason, together with aggression toward others, that individuals are either retained in institutional (restrictive) environments or are administered psychotropic medication. Today, SIB remains unmanageable, expensive ($150,000–$500,000/year/patient in institutional settings, with national costs well over $3,000,000,000 [1]), and is often life threatening. It is surprisingly prevalent, occurring in ∼30% of individuals with developmental neurological complications, including those with autistic disorders [2,3].

**Figure 1:**
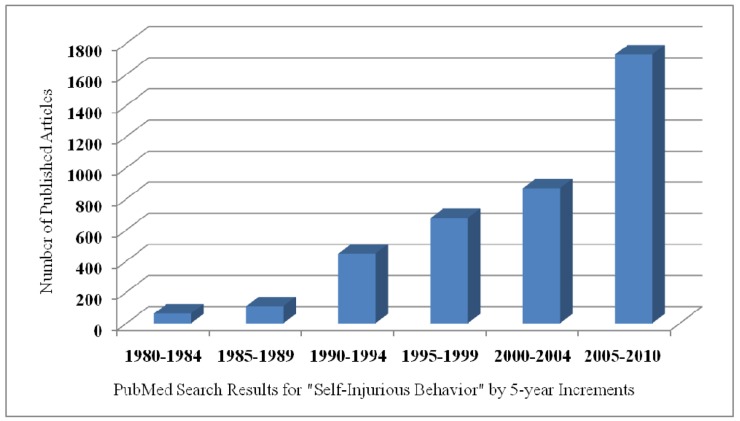
Number of studies of self-injurious behavior (SIB) conducted in 5-year intervals from 1980 to the present.

Twenty-five experts invited by the National Institute of Child Health and Human Development (NICHD) to discuss the relations among the genetic, neurobiological, and behavioral causes and treatments for SIB reached two general conclusions. Intentional acts of harm to self, evident in many species, (a) have no known cause and (b) no agreed upon treatment [4]. The apparent absence of visible progress in understanding or treating SIB is not because of a lack of interest or effort. Studies of self-injury have increased exponentially over the past 30 years, rising from just 60 published studies between 1980 and 1984 to over 1,700 studies reported between 2005 and 2010 (Figure 1).

One major obstacle in understanding the mechanisms of SIB and developing coherent treatment plans is the absence of distinctive behavioral phenotypes. Despite the consensus that SIB has variable expression with no known cause, the group of NICHD experts agreed that SIB could be defined, perhaps with greater precision than most complex human behaviors. SIB is a directly observable behavior that can be reliably counted. The NICHD group argued that data collection and analysis had advanced so that complex patterns of SIB should replace or supplement measures of rate and frequency [5,6]. Two distinct patterns of SIB were proposed as possible guides. One pattern consists of bouts that are most likely maintained by environmental contingencies. The second pattern involves protracted periods of SIB that are most likely under the primary influence of biological factors. The vast majority of existing studies, however, have reported frequencies or rates of occurrence of SIB often linked to a single environmental manipulation. It is a significant advantage that many forms of SIB can be counted and time-stamped enabling contemporary studies to use sequential and time series procedures to define their structure and their relations with other behaviors and the environment. We subjected extensive and lengthy observations of maladaptive behavior in its natural environmental context to time series analysis and discovered unique temporal and sequential patterns of these severe maladaptive behaviors [2,3,7-10].

Specifically, we found for a large majority of the individuals studied, SIB was predicted only by its own recent history. In our cohort of severely self-injurious patients, SIB was expressed consistently in successive occurrences revealing a unique pattern of sequential dependence compatible with a “contagious” distribution. Application of time-series methods of analysis that controlled for chance pairings of events indicated that the contagious patterns of SIB were independent from frequency and rate of occurrence. That is, the temporally dependent patterns we observed were not a function of high rates of occurrence. Moreover, and surprisingly, SIB was not associated consistently with other behaviors or with the several staff activities or environmental conditions recorded in these studies. Thus, in a significant majority of these individuals, SIB episodes were self-perpetuating and not related to antecedent or subsequent environmental circumstances, events, or other recorded behaviors.

This novel and surprising finding was consistent with conclusions of the NICHD group that some expressions of SIB may have an underlying biological basis because a solely self-perpetuating behavior is most parsimoniously explained by internal (*i.e.*, biological) motives [2,11-13]. The vast majority of individuals in our cohort exhibited the most primitive level of internally regulated behavioral patterns despite years of behavioral interventions and treatment with various medications [8,9]. Anecdotal and clinical observations of these individuals also strongly suggested a biological basis for their behavior and specifically involvement of the pain and pleasure systems. Typically, SIB is repetitious consisting of hourly, daily, weekly, monthly, or even yearly cycles [14]. Some individuals who repeatedly injure themselves appear immune to the normal experience of pain [12]. They abuse and injure their bodies, hitting or biting themselves, hurling themselves to the ground, and banging their head against solid objects resulting in broken bones, disfigurement, blindness, and even loss of life [2,15,16]. They often work to overcome interventions designed to decrease self-injury in a manner that is consistent with seeking positive reward. For instance, protective devices (such as helmets) may result in individuals exerting greater effort and exhibiting greater rates of behavior to hit and harm themselves [17].

Because many medications that treat pain or induce pleasure are addicting, it is interesting that SIB, in addition to the obvious involvement of pain systems, shares features of addiction such as compulsive and ritualistic (or stereotypic) patterns that either comprise or surround the self-injuring acts. One biological system that has been implicated in SIB and the modulation of pain and pleasure is the hypothalamic-pituitary-adrenal (HPA) stress axis and specifically the proopiomelanocortin (POMC) molecule [3,18,19].

## 2. The Biological Stress System Pain, Pleasure, and SIB

Thompson and Caruso [6] recognized that some forms of SIB were “neurochemically driven and independent of environmental events.” Early studies either of basal or of resting levels of a variety of peptides, proteins, transmitters, and amines in plasma or cerebrospinal fluid from patients exhibiting SIB, however, generated inconclusive results. This was not surprising because there was little consistency among studies regarding the rigor of diagnosis, the molecule measured, the tissue assayed, or the conditions assessed [20-26].

### 2.1. Pain and the Endogenous Opioid System

Although it is not a universal observation, most self-injurious individuals do not exhibit the usual signs of pain after their “injurious” behavior. Despite inflicting serious physical damage to their bodies, many of these individuals do not grimace, cry, or show other symptoms that they are experiencing pain. It has been suggested that this absence of response to self-inflicted injury reflects insensitivity to pain and general sensory depression induced either by elevated endogenous opiates or by supersensitive opiate receptors [11,27-29]. This possibility is supported by classical findings that opiate receptor blockers (a) reverse congenital insensitivity to pain [30]; (b) normalize hypothalamic-peptide dysfunction coexisting with elevated pain threshold [31]; and (c) increase brain responses to sensory information [32]. These observations are consistent with a venerable animal literature proving that opiate blockers lower pain threshold [33]. In summary, these findings support an analgesia (or pain) hypothesis that implies that self-injurious individuals do not feel pain because of *chronically* elevated endogenous opiates and/or opiate receptor downregulation.

### 2.2. Pleasure (Addiction) and the Endogenous Opioid System

It also is possible that the addictive properties of elevated endogenous opiates are responsible for maintaining SIB. If it is presumed SIB does result in pain and that the experience of pain results in the release of opiates, then it can be argued that individuals commit self-inflicted harm to receive the euphoric (pleasurable) effects of increased circulating opiates. From this perspective, SIB is an “addiction” to the endogenous opiate system because its consequences supply a “fix.” It has been known for over 25 years that endogenous opiates have addictive properties as indicated by the development of tolerance [34], physical dependence [35], and euphoric-like effects [36] after repeated administration. The repetitive, often compulsive, and ritualistic patterns of SIB (*i.e.*, injury to one area of head or body, stereotyped patterns of behavior, and catastrophic responses if the environment is slightly changed) are similar to rituals and compulsive patterns often associated with addictive behaviors. The addiction hypothesis maintains that individuals with SIB may endure the pain to enjoy the pleasure it produces as well as to avoid a withdrawal effect. The addiction hypothesis predicts that SIB may be reinforced both positively and negatively because it gains the individual access to the narcotic effect of endorphins while simultaneously allowing the individual to escape the unpleasant sensory consequences commonly associated with the absence of opiates following chronic and sustained access.

### 2.3. Stress and the Endogenous Opioid System

The endogenous opioid system is tightly coupled with the general stress response. Evidence from several laboratories indicates that functioning and processing of a stress-related molecule (POMC) in the HPA axis may be perturbed among subgroups of individuals exhibiting SIB [15,19,21,37-43]. In humans, most POMC is produced in the pars distalis of the anterior pituitary but also by hypothalamic neurons and neurons in the amygdala and pituitary stalk. POMC is a well-characterized 31-K dalton, bioinactive protein-like molecule that is post-translationally converted by enzymes (e.g., PC1 and PC2) into biologically active fragments, including B-endorphin (BE) and adrenocorticotropic hormone (ACTH) [15,44-48]. Normally, BE is coreleased from the anterior pituitary with ACTH in response to a variety of stressors. However, elevated BE but not ACTH is associated with SIB either at rest or after an episode of SIB [3,12,18]. This suggests that one consequence of SIB is the disregulation of the arousal system. The validity of these hypotheses is not known but they have encouraged treat-ments, including opiate receptor blockers, designed to regulate the opiate/stress system as means to control SIB.

## 3. Efficacy of Opiate Blockers in the Treatment of SIB

### 3.1. Acute Effects of Naltrexone

In a review of pre-1991 studies [49], six of eight published studies reported that injectable naloxone significantly reduced SIB. In these eight studies, naloxone was tested in a total of ten individuals with SIB. A decrease in SIB was reported for seven individuals. In that same review, 12 published studies of naltrexone (Naltrexone) in MR/DD individuals were summarized. Most of the studies either were case studies or were studies with very small samples. At that time, 45 MR/DD individuals (at least 28 with SIB) had been treated with naltrexone and 38 individuals had positive responses of various degrees including a reduction in SIB in 24 of the 28 patients. A separate review of 13 studies (including several in the Sandman review [49]) concluded that about one-third of the patients tested with Naltrexone had a decrease in their SIB [50]. Several studies in this later review included juvenile patients under the age of 8 years [51] and patients with primary behavioral problems related to aggression and agitation [52]. Aggression toward others and agitation are not equivalent to SIB on any obvious dimension except, perhaps, exertion, and the fact that opiate blockers were ineffective in the control of these behaviors adds inferential support to the argument that the opioid system is uniquely implicated in SIB and not in other maladaptive behaviors. The effects of opiate blockers in children who self-injure may be similar to the effects observed in adults but there are too few reports to make that conclusion.

More recently, a thorough review of the scientific literature employing stringent and appropriate criteria for inclusion concluded that the effects of opiate blockers on SIB could be evaluated in a total of 86 patients [53]. Eighty percent of the subjects were reported to improve relative to baseline (*i.e.*, SIB reduced) during naltrexone administration. Of the subjects who improved, 47% exhibited a reduction in SIB by 50% or greater. In studies reporting dose levels in milligrams, males were more likely than females to respond. No significant relations were found between treatment outcomes and autism status or form of self-injury.

Two relatively large, placebo-controlled studies [16,54] included in this review of naltrexone came to very similar conclusions. In a double-blind, placebo-controlled, dose-finding study, Sandman *et al.* [54] reported that 18 of 21 individuals exhibiting SIB responded favorably to at least one dose (range of 0.5–2.0 mg/kg) of naltrexone (time-sampled video records provided direct observations of the subjects). Acute treatment (1 week at each of three doses) with naltrexone reduced the frequency of SIB without major side-effects. Activity, stereotypy, involuntary movement, and neurological status were not influenced by naltrexone. There were two central findings. First, the highest dose (2 mg/kg) was the most effective, confirming earlier results in this population [55,56]. Seven of the eight patients responding best at the highest dose, also responded favorably to the 1 mg/kg dose. Six of these eight patients also responded at the 0.5 mg/kg dose. Eleven subjects responded positively to both the 1 and 2 mg/kg doses. Second, subjects with the most frequent SIB were the most positive responders to higher doses of naltrexone, consistent with earlier reports [55,56]. A small minority of subjects responded most favorably to lower doses. These results confirmed that at least 50% of the individuals with SIB responded favorably to treatment with opiate blockers.

Another double-blind, placebo-controlled, fixed-dose study of eight, severe to profoundly retarded adults included in the review [16], reported that treatment with naltrexone reduced head hitting, head banging, and self-biting. The eight individuals evaluated displayed 18 forms of SIB. Improvement was observed in 77% of the head hitting and head banging episodes and 100% of the self-biting forms. Episodes of high frequency SIB also were more sensitive to treatment with naltrexone. The 100-mg (high) dose was more effective than the 50-mg (low) dose in reducing SIB. For several individuals, some forms of SIB decreased after naltrexone (e.g., head hitting and self-biting) but other forms (e.g., throat poking) did not change. Four of the subjects in this trial received concomitant treatment with clonidine (alpha-2-adrenergic agonist) but no effects on SIB or interactions with naltrexone were observed. These findings compliment previous studies and caution that although naltrexone is effective in reducing SIB, not all forms of self-inflicted harm may be controlled by blocking the opioid system.

These two relatively large studies of *acute treatment* with naltrexone came to very similar conclusions. Opiate blockers appear to be an effective treatment for a significant number of individuals exhibiting SIB. Administration of naltrexone reduces high frequency SIB and some, but not all, self-destructive behavior. Both studies acknowledged that not all individuals expressing SIB were positive responders and that a small minority may increase SIB (see also Barrett [57]).

In the single study that has evaluated the effects of naltrexone using time-series analysis, Symons *et al.* [58] made very interesting observations. First, they reported that three of the four patients evaluated had at least a 33% reduction in their SIB. (The fourth patient had a 17% reduction in SIB.) Second, and most interesting, they discovered that in addition to the improvement with naltrexone, there was an alteration in the sequential dependencies between staff behavior and the manifestation of SIB. During treatment with naltrexone, there was a significant increase in the probability that staff would “prompt” individuals proximal in time and sequence to an SIB event for three of the four patients, and a significant decrease in the fourth. One possible conclusion from these findings is that naltrexone exerts its effects on SIB, in part by the opioid-mediated reinforcing influences of social interactions. Alternatively, Symons *et al*. [58] suggest that SIB may be “multiply determined such that naltrexone may diminish opiate-mediated SIB leaving socially mediated SIB unchanged.” The overall clinical implication is that many cases of SIB may be subserved by both opiate- and socially mediated processes and that effective treatment for such cases would require a combination of naltrexone with behavioral intervention strategies.

### 3.2. Long-Term Effects of Naltrexone Treatments

The long-term effects and consequences of continued treatment with naltrexone is not completely known because most studies reporting treatment of individuals with SIB have been short-term demonstrations or acute trials. Most published long-term studies have been either case studies or open-label designs and they have generated mixed results. Two types of studies comprise the long-term evaluations of naltrexone, either prolonged treatment with naltrexone or extended observations following brief periods of treatment. With these procedures, investigators have reported that about six of eight patients examined in several studies exhibited long-term benefits in varying degrees from treatment with naltrexone [57-59]. In the first report, a total of 24 days of naltrexone treatment resulted in elimination of SIB in a 12-year-old girl that persisted for at least 22 months [57]. A similar finding was reported after 1 year of continuous treatment with naltrexone in a 28-year-old woman with severe SIB. Not only did treatment eliminate SIB but also the near-zero rate persisted through placebo and no-drug phases of the study [59]. In their retrospective study of 56 patients, Casner *et al.* [60] discovered that 57% of their patients treated with naltrexone between 3 and 878 months were considered to be positive responders and 25% of these met objective criteria as responders.

We [61] examined the long-term (12 month) effects following acute treatment with naltrexone and then we assessed the effects of subsequent long-term treatment with naltrexone. To accomplish this, we enrolled 15 subjects in a double-blind, placebo-controlled acute dose-finding study. Each acute dose was evaluated for a 1-week period with placebo weeks interspersed. Subjects were followed for a 12-month period and then they were enrolled in a multiple baseline design with a single most effective dose (determined in the acute phase) administered to each subject for 2-, 3-month periods over an 18-month interval with placebo periods appropriately separating the treatment phases. Again, time-sampled video records were scored using a computer-assisted program [62].

The primary finding from our study was that a subgroup of patients exhibited persisting effects (decreased SIB) in the 12 months after acute treatment with naltrexone. Seven patients exhibited decreased SIB over the 12-month period and five of these had a 75% reduction in SIB compared to the placebo control period. These five patients, each with at least a 75% reduction in SIB, increased their SIB when administered naltrexone in the long-term treatment protocol. The largest decrease in SIB was observed in patients who had a brief exposure to naltrexone, were given a 12-month hiatus during which they showed an increase in SIB, and then were readministered naltrexone several times in the 18-month double-blind, placebo-controlled study.

## 4. Endogenous Opioid Levels Predict Response to Opiate Blockers

In our initial study to examine the relation between circulating endogenous opioids and response to naltrexone, we collected blood samples from ten patients within 2–5 min of a self-injuring act and during a control period [18]. At least 1 month later patients were administered three different doses of naltrexone in a double-blind, placebo-controlled crossover study over a 10-week period. All patients were videotaped during the study and behavior was coded with a computer-assisted program. Patients with the highest change in plasma levels of BE after SIB had the most and statistically significant positive response to naltrexone. These results were consistent with several other reports. First, Ernst *et al.* [21] reported that baseline levels of BE were positively related to changes in behavior (clinical global impressions, CGI) after treatment with naltrexone in five young autistic children. Second, Bouvard *et al.* [38] found that C-terminal BE decreased after naltrexone only in good responders. Third, Scifo *et al.* [63] found that increases in SIB and response to naltrexone in some patients, were related to high levels of endogenous opiates (*i.e.*, good responses to naltrexone were observed in patients with high levels of BE). Fourth, Cazzullo *et al.* [64] reported that patients responding with decreased BE levels after treatment with naltrexone had better and more pervasive behavioral improvement than patients who did not have physiological changes after naltrexone.

In a follow-up study of nine additional patients (total of nineteen), we [3] found that plasma BE was uncoupled from the usually coreleased ACTH [65-71] after an episode of SIB. This unusual pattern was not a function of time of day that blood was sampled, and it confirmed our earlier observations [7,18] and provided additional support for this specific biological marker among a diverse group of subjects who share a behavioral aberration. In addition, stronger support was generated for the effectiveness of naltrexone in reducing SIB.

Positive responses to low doses of naltrexone were observed in subjects *who did not* exhibit increased BE after SIB. That is, low doses of naltrexone were effective in reducing SIB only in subjects either who did not exhibit a surge in BE after SIB or whose baseline level exceeded the level after SIB. The relation between BE and response to the lowest dose of naltrexone was consistent with our earlier results, and statistically significant with the addition of nine subjects. Previously we [3] suggested that SIB had functional significance because it increased endogenous opiates and thereby delivered positive consequences (*i.e.*, pleasure/euphoria/pain modulation). We argued that the highest dose of naltrexone most effectively blocked this mechanism in subjects with the highest levels of BE after SIB. The results from the follow-up study suggested an alternative possibility related to baseline levels of, or baseline relations between, POMC peptides. Because the lowest dose of naltrexone was most effective in subjects with the highest (morning) baseline (relative to post-SIB) levels, we speculated that the association between baseline ACTH and BE could influence the response to naltrexone (based on evidence that supported reciprocal functions of BE and ACTH [72]]. If our speculations were accurate, subjects with the greatest difference between morning BE and ACTH levels would be the most responsive to low doses of naltrexone because there would be less attenuation of the opioid influence. The test of this possibility confirmed our speculations because we found that subjects with high levels of morning (chronic) BE *and* low levels of ACTH were associated with positive responses to the low dose of naltrexone. This possibility may be compatible with findings that chronic exposure to opioids resulted in supersensitivity to the effects of low doses of opiate antagonists [73,74].

These findings have since been extended using a fundamentally different method for quantifying changes in the temporal patterning of SIB following treatment with naltrexone. The THEME method developed by Magnusson [75,76] has been used to detect highly significant, nonrandom, hierarchical patterns in the temporal organization of SIB with respect to other observed behaviors. Kemp *et al.* [10] reported that these temporal patterns (“T-patterns”) of SIB were significantly correlated with basal levels of BE. In the subgroup of subjects receiving naltrexone (discussed above), the percent change in these T-patterns of SIB (between weeks the subjects were receiving placebo and weeks they were receiving naltrexone) was found to be significantly correlated with post-SIB (*i.e.*, samples were collected immediately after SIB) levels of the N-terminal fragment of beta-endorphin (βEN). No such correlations were found for changes from placebo on any “control” T-patterns (those containing non-injurious, stereotyped behaviors or staff interactions) nor for any samples collected during other periods. These results are shown in Table 1.

We have made similar observations in our long-term studies of naltrexone and SIB [19]. POMC fragments were measured in twelve self-injurious patients before and after long-term (3-month) treatment with naltrexone. POMC fragments were sampled from blood collected at the end of the baseline and placebo-controlled treatment phases of the study. Two patterns emerged. One group (responders) displayed persisting improvement in SIB and lower relative levels of BE after initial exposure to naltrexone. Chronic administration of naltrexone to this group was associated with increased SIB and elevated relative levels of BE. Return to placebo improved their behavior (reduced SIB) and their levels of BE returned to basal levels. The second group (nonresponders) was characterized by absence of persisting improvement after acute treatment with naltrexone and by elevated basal BE levels. Chronic treatment with naltrexone improved their behavior but did not alter their BE levels. Long-term positive responders to acute doses of naltrexone were associated with less disregulation of ACTH and BE.

## 5. Conclusions

When it was established that the body had its own opiate system [77], the endogenous opiates became prime suspects responsible for maintaining SIB. Perhaps individuals who self-injure have elevated thresholds for pain or derive pleasure from painful stimulation. Exposure to, or levels of, endogenous opiates could explain these possibilities. Reduction in SIB following treatment with opiate blockers would provide evidence for the opiate hypothesis of self-injury. Results from studies to test these possibilities, however, are complex. The complexity is related primarily to the fact that patients exhibiting SIB and evaluated after treatment comprise a mixture of etiologies, pathologies, and motivations. Despite the tremendous amount of error introduced with a heterogeneous population, there is substantial evidence that opiate blockers are efficacious in reducing SIB.

The observations that opiate blockers reduce SIB are important for at least two reasons. First, naltrexone produces a clinically significant reduction in a serious and life-threatening behavior for some individuals typically who have not been responsive to any other type of treatment. We have observed startling improvements in individuals who have failed all rational treatments. Some adults in our studies have had protective headgear discontinued for the first time since early childhood. Others have developed adaptive skills and have acquired the ability (or the privilege) to leave institutions for the first time in their lives after treatment with naltrexone. Second, the results with naltrexone are important because they suggest that a *specific biological system* may be disregulated in a subgroup of patients. Because the opiate blockers have few effects in the absence of opiates [78], effective treatment with these drugs must engage the endogenous opioid system. Reports that resting levels of endogenous opiates or levels of endorphin after an SIB episode predict positive responses to opiate blockers provide support for this assumption and the foundation for rational treatment strategies based on biological criteria.

From the current review we can draw several conclusions. There is consensus in the literature that doses between 1.0 and 2.0 mg/kg or a fixed dose of 100 mg are the most effective for reducing SIB [16,53,54]. At these doses, at least half of the chronically self-injuring patients exhibit at least a 25% reduction in their behavior. It is important to acknowledge that the studies that have reported reductions of 25% or greater, typically conducted direct observations of the patients and did not rely on global clinical ratings. As reviewed by Symons *et al.* [53], male patients respond more favorably to naltrexone treatment than do female patients. There is consensus that naltrexone is a safe drug without major or contraindicating side effects. Most individuals entered into naltrexone trials exhibited the most severe SIB for which all other forms of treatment had been ineffective. Often their behavior presented life-threatening consequences and *always* their SIB prevented them from enjoying a less restrictive environment. Against this background, the possible benefits of reduced SIB by treatment with naltrexone exceed the risk of side-effects. We are aware of clinical decisions to treat SIB with naltrexone even when patients presented with risk factors such as chronic hepatitis [79]. To our knowledge, there have not been serious side effects *solely* due to administration of naltrexone among MR/NDD patients. There is consensus among the studies that naltrexone is an effective treatment because the endogenous opioid system is engaged by SIB [3,16,18,19,57]. SIB has characteristics that resemble addictive behavior (compulsive, ritualistic, destructive) and altered pain threshold. Both of these characteristics implicate the opioid system and support the logic of opiate blockers as reasonable treatments. There is consensus that long-term treatment of SIB with naltrexone apparently is not harmful and may be effective. There is consensus that generally, treatment with naltrexone appears to be effective in about half of the adult patients examined. Following effective acute treatment with naltrexone some patients have shown a rebound to pre-treatment levels [61]. There is consensus that naltrexone should be avoided during periods when patients are known to be in pain requiring narcotic analgesics, such as surgeries or with bone fractures. Alternately, nonnarcotic analgesics should be administered to patients receiving naltrexone [80]. Finally, it should be noted that there are reports of paradoxical increases of SIB following treatment with naltrexone [81]. In our own research [61], we have observed increased rates of SIB in individuals who showed sustained improvements throughout a 12-month hiatus following a brief period of acute treatment with naltrexone, and then were given longterm treatment (3 month) with naltrexone.

## Figures and Tables

**Table 1:** Pearson's r coefficients (significance) between levels of beta-endorphin (N-terminal) collected in either the AM, PM, or following SIB, no SIB, or physical exercise (PE) and the change in the percentages of T-patterns (by behavior type) for weeks treated with placebo and naltrexone.

	**Beta-Endorphin (N-terminal) Levels**				

Change in T-Pattern % by Type:	**AM**	**PM**	**Post-SIB**	**No-SIB**	**Post-PE**
**SIB**	0.37 (0.47)	0.25 (0.64)	0.82 (0.04)	−0.45 (0.44)	0.66 (0.22)
**Stereotypy**	−0.76 (0.07)	−0.26 (0.96)	0.52 (0.29)	−0.29 (0.64)	−0.10 (0.87)
**Staff Interactions**	0.42 (0.41)	0.54 (0.27)	0.35 (0.49)	−0.63 (0.25)	0.26 (0.68)
